# Identifying GPCR-drug interaction based on wordbook learning from sequences

**DOI:** 10.1186/s12859-020-3488-8

**Published:** 2020-04-20

**Authors:** Pu Wang, Xiaotong Huang, Wangren Qiu, Xuan Xiao

**Affiliations:** 1 Computer School, Hubei University of Arts and Science, Xiangyang, 441053 China; 2 Computer Department, Jingdezhen Ceramic Institute, Jingdezhen, 333403 China

**Keywords:** GPCR-drug interaction, Bag-of-words, Discrete Fourier transform, Machine learning

## Abstract

**Background:**

G protein-coupled receptors (GPCRs) mediate a variety of important physiological functions, are closely related to many diseases, and constitute the most important target family of modern drugs. Therefore, the research of GPCR analysis and GPCR ligand screening is the hotspot of new drug development. Accurately identifying the GPCR-drug interaction is one of the key steps for designing GPCR-targeted drugs. However, it is prohibitively expensive to experimentally ascertain the interaction of GPCR-drug pairs on a large scale. Therefore, it is of great significance to predict the interaction of GPCR-drug pairs directly from the molecular sequences. With the accumulation of known GPCR-drug interaction data, it is feasible to develop sequence-based machine learning models for query GPCR-drug pairs.

**Results:**

In this paper, a new sequence-based method is proposed to identify GPCR-drug interactions. For GPCRs, we use a novel bag-of-words (BoW) model to extract sequence features, which can extract more pattern information from low-order to high-order and limit the feature space dimension. For drug molecules, we use discrete Fourier transform (DFT) to extract higher-order pattern information from the original molecular fingerprints. The feature vectors of two kinds of molecules are concatenated and input into a simple prediction engine distance-weighted K-nearest-neighbor (DWKNN). This basic method is easy to be enhanced through ensemble learning. Through testing on recently constructed GPCR-drug interaction datasets, it is found that the proposed methods are better than the existing sequence-based machine learning methods in generalization ability, even an unconventional method in which the prediction performance was further improved by post-processing procedure (PPP).

**Conclusions:**

The proposed methods are effective for GPCR-drug interaction prediction, and may also be potential methods for other target-drug interaction prediction, or protein-protein interaction prediction. In addition, the new proposed feature extraction method for GPCR sequences is the modified version of the traditional BoW model and may be useful to solve problems of protein classification or attribute prediction. The source code of the proposed methods is freely available for academic research at https://github.com/wp3751/GPCR-Drug-Interaction.

## Background

As the largest family of human membrane protein, GPCRs mediate multiple physiological processes such as neurotransmission, cellular metabolism, secretion, cellular differentiation, growth, inflammatory, and immune responses [1, 2]. As a result, these receptors have emerged as the most important drug targets in human pathophysiology [3, 4]. According to the report in [5], 475 drugs target 108 unique non-olfactory GPCRs, this account for about 34% of all drugs approved by the US Food and Drug Administration (FDA). Furthermore, dozens of novel GPCR targets that are not yet modulated by approved drugs are now in clinical trials, these receptors are potentially novel targets for the treatment of various indications.

The GPCR-related drug discovery often relies on the binding affinity identification. The traditional high-throughput screening (HTS) method is receptor binding assay, such as scintillation proximity assay (SPA) and time-resolved fluorescence resonance energy transfer (TR-FRET) technology [6]. However, with the development of computational methods for GPCR drug discovery, the HTS can be aided by in silico modeling, including the structure-based methods and the sequence-based methods. The combination of in vitro and in silico methods will reduce both time and cost by reducing the number of candidate compounds to be experimentally tested. The structure-based approach plays an important role in drug discovery, especially for enzyme-targeted drugs [7, 8]. However, this approach is restrained in the development of GPCR-targeted drugs because it is very difficult to acquire the reliable 3D structures of these receptors. With the breakthroughs in GPCR crystallography [9, 10], the structure-based methods are potentially impactful for GPCR-targeted drug design [11–13]. As far as it goes, sequence-based methods may be an easy and efficient choice owing to machine learning technology and the accumulation of target-drug interaction data stored in KEGG [14], SuperTarget [15], DrugBank [16] and so on.

Identifying the target-drug interaction has become a hot topic in bioinformatics, and a great deal of effort has been made in this area to bring up many effective methods [17–26]. Because the importance and particularity of GPCRs (the available 3D structures are very limited), we focus the study in the computational approach for identifying GPCR-drug interaction only based on sequence information.

Because there are two types of molecules involved in the interaction between GPCRs and drugs, the method of combining the chemical structure information of drugs and sequence information of proteins is often used here. Yamanishi et al. [27] did a series of research in the prediction of target-drug interaction networks, including the GPCR-drug interaction. The methods used in these studies are in fact based on sequence similarities, including the chemical structure similarities between compounds computed by SIMCOMP [28], the pharmacological effect similarities between compounds computed by the weighted cosine correlation coefficient, and the sequence similarities between the proteins computed by a normalized version of Smith–Waterman scores [29]. And then in the framework of supervised bipartite graph inference, compounds and proteins were mapped onto the unified feature space, in which the more closer the compound and protein were, the more likely that two objects interacted with each other. Differently, He et al. [30] studied the GPCR-drug interaction based on functional groups and biological features. In this method, any drug was formulated as a 28D feature vector according to its chemical functional groups, and any protein was formulated as a 139D feature vector using pseudo amino acid composition (PseAAC) [31, 32] method. And then machine learning technology such as feature selection and the nearest neighbor algorithm were adopted to solve the problem of interaction prediction. iGPCR-Drug [33] was also a sequence-based method specifically proposed for identifying GPCR-drug interaction. In this method, any drug was represented as 2D fingerprint via a chemical toolbox called OpenBabel [34], and then DFT was used to extract 256D frequency features for each drug. Accordingly, any GPCR was formulated as a 22D feature vector through PseAAC method. In such a way, any GPCR-drug pair, no matter interaction or non-interaction, could be formulated as a 278D feature vector by combining the two types of feature vectors. Finally, these feature vectors were input into the fuzzy K-nearest-neighbor classifier for interaction recognition. Recently, Hu et al. [35] proposed a new sequence-based method for the prediction of GPCR-drug interaction. In this method, the discrete wavelet transform (DWT) was utilized to extract the features of drugs based on their fingerprints, and any drug molecular was represented as a 128D feature vector. For GPCRs, the pseudo position specific scoring matrix (PsePSSM) features were extracted to encode any GPCR as a 140D vector. With the combined 268D feature vectors as input, several classifiers were tested, including optimized evidence theoretic K nearest neighbor (OET-KNN), radial basis function networks (QuickRBF), support vector machine (SVM), and random forest (RF). The experiment results showed that RF performs better than the others consistently. To reduce the false positive and false negative errors, the initial model was further improved with a drug-association-matrix-based PPP. Although this advanced model characterized by the combination of progressive feature extraction method (PsePSSM and DWT), ensemble learning method (RF), and post-processing procedure (PPP) was better than the foregoing ones, but it seemed that the generalization ability of this advanced model was still limited because the results of independent test were much lower than that of cross-validation on training dataset, especially when there was no PPP. So it is very meaningful to develop models with high generalization.

In this study, we propose a new powerful sequence-based method for identifying the GPCR-drug interaction based on wordbook learning from sequences. For GPCRs, we encode the sequences by the physicochemical properties of amino acids, and then create a wordbook through clustering technology. Based on the wordbook, any GPCR is formulated as a feature vector containing the word frequencies. It is easy to construct the wordbook of drugs by carrying out DFT on the drugs’ fingerprints and the amplitude compositions at different frequency points are taken as the words of the drug wordbook, and then each drug is formulated as a feature vector equivalent to the dimension of GPCR feature vector. In the joint feature space of GPCR-drug pairs, a very simple machine learning method, DWKNN [36], is employed as the classifier for interaction prediction. This basic method is very easy to be enhanced through ensemble learning. Independent test on the benchmark dataset demonstrates the generalization ability of the proposed methods.

## Results

Firstly, we fix the hyperparameter in the prediction engine DWKNN, and compare the prediction performance with different representations of GPCRs and drugs. Secondly, with the best feature representation proved by experiments, we test the effect of hyperparameter in DWKNN. Thirdly, we try to enhance the base model through ensemble learning. Finally, we compare the performance of the proposed methods with that of the previous ones through cross-validation and independent test.

### Experimental datasets and performance measurement

Our experiments are carried out on two recently constructed datasets: D92M and Check390 [35], which are used for training and independent test respectively. D92M contains 92 unique GPCRs and 217 unique drugs, and they constitute 635 interactive pairs and 1225 non-interactive pairs. D92M is in fact a refined dataset based on the original data used in [30] by correcting the falsely labeled GPCR-drug pairs. Check390 consists of 130 interactive pairs and 260 non-interactive pairs that do not appear in the training dataset. The metrics for performance evaluation used in our experiments include Receiver Operating Characteristic curve (ROC), Area Under an ROC Curve (AUC), Sensitivity(Sn), Specificity (Sp), Strength (Str, the average of Sensitivity and Specificity), Accuracy (Acc), and the Matthews correlation coefficient (MCC).

### Effect of different physicochemical properties for encoding GPCRs

**Fig. 1:**
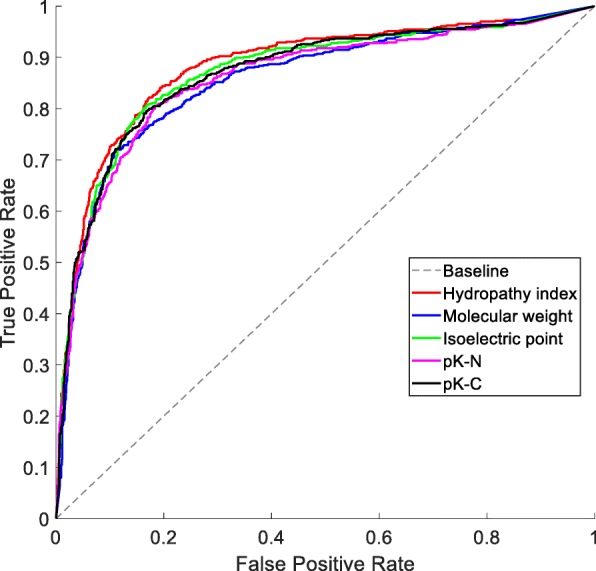
ROC curves of ten-fold cross-validation on D92M with different amino acid indices for encoding GPCRs

There are more than 500 amino acid indices in AAindex, which is a database of numerical indices representing various physicochemical and biochemical properties of amino acids and pairs of amino acids [37]. In this section, we test the effects of five common amino acid indices: hydropathy index (Entry: KYTJ820101), molecular weight (Entry: FASG760101), isoelectric point (Entry: ZIMJ680104), pK-N (Entry: FASG760104) and pK-C (Entry: FASG760105). The ROC curves of ten-fold cross-validation on D92M with different amino acid indices for encoding GPCRs are shown in Fig. 1. Because hydropathy index has the biggest AUC, we choose it as the default amino acid index.

### Effect of different feature representations of drugs

**Fig. 2:**
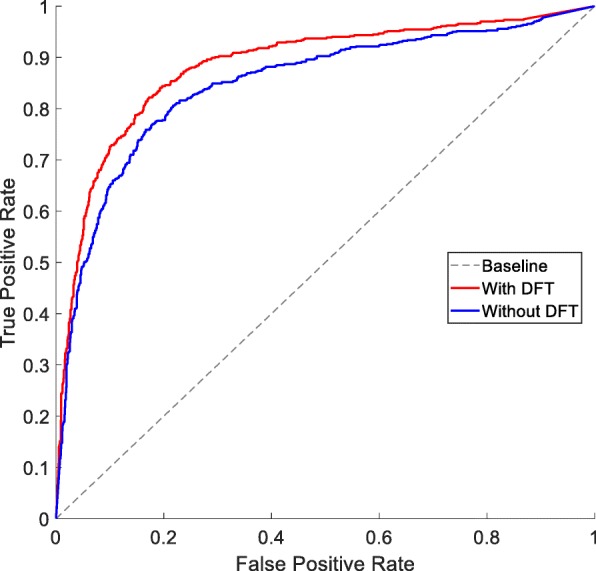
ROC curves of ten-fold cross-validation on D92M while representing drugs with primary molecular fingerprint (without DFT) or frequency amplitudes (with DFT)

Though DFT was successfully used in previous work [33], no contrast experiment was carried out to prove the necessity of DFT. In this section, with the fixed GPCR representation, ten-fold cross-validation is carried out on D92M while representing the drugs with primary molecular fingerprint (without DFT) and frequency amplitudes (with DFT) respectively, and the ROC curves are shown in Fig. 2. It is clear that the ROC curve of DFT is always above that of without DFT. This is because the DFT can extract more pattern information than original structural description in the form of molecular fingerprint.

### Effect of different feature representations of GPCRs

**Fig. 3:**
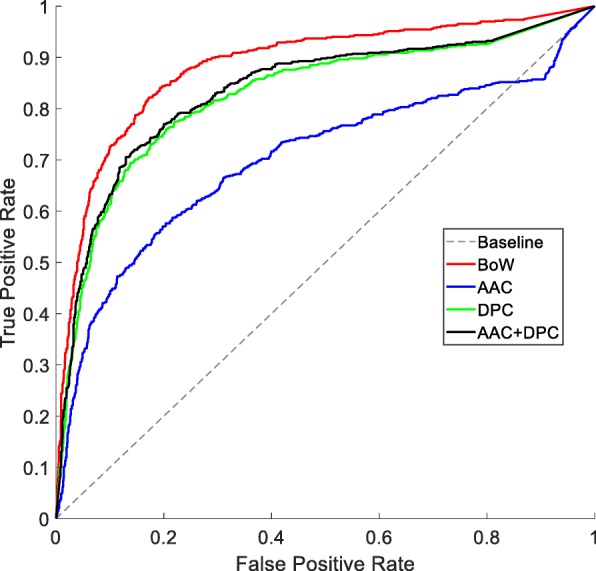
ROC curves of ten-fold cross-validation on D92M with different feature representations of GPCRs

In this experiment, we compare the proposed BoW model with the traditional ones like amino acid composition (AAC), dipeptide composition (DPC), and their combination AAC + DPC. Figure 3 shows the ROC curves of different feature representations of GPCRs through ten-fold cross-validation on D92M. It can be found that the performance of AAC is the worst. This is inevitable because the sequence order information is completely ignored. By taking the adjacent information into account, the results of DPC are better than AAC. The performance of AAC + DPC is only slightly better than DPC. The performance of the proposed method is significantly better than the others, because more sequence order information and physicochemical information are taken into account.

### Effect of hyperparameter in DWKNN

**Fig. 4:**
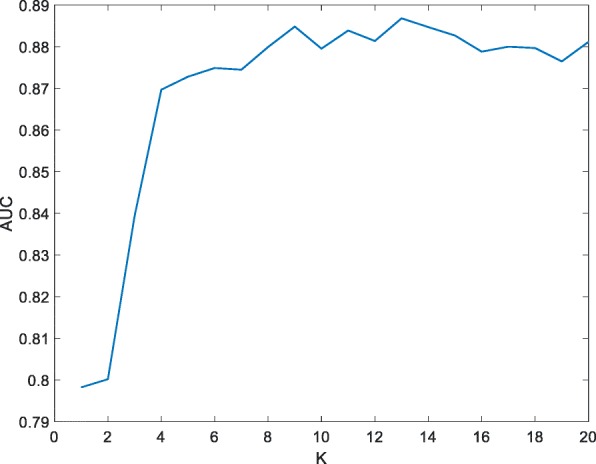
AUCs of ten-fold cross-validation on D92M with different K values in DWKNN

Figure 4 shows the AUC values while using different *K* values in DWKNN. As we can see, at the beginning, AUC is improved significantly along with the increasing of nearest neighbors. However, after *K* = 8, its values begin to oscillate, and when *K* = 13, it reach maximum. So *K* = 13 is set as the default hyperparameter value in DWKNN when using hydropathy index for GPCRs and DFT for drugs.

### Effect of hyperparameter in ensemble model

In Fig. 1, the ROC curves with different amino acid indices don’t look very different from each other, and what will happen if all the five indices are used? In this experiment, we try to enhance the base model through Bagging, which is the ensemble learning method used by RF [35]. In the proposed ensemble model, the number of prediction engines for each amino acid index (called *N*_e_) is the only hyperparameter, and its impact on the ensemble model is displayed in Table 1, from which we can find three points. Firstly, compared with the best base learner (use hydropathy index and DWKNN engine with K = 13), nearly all the metrics are improved in the ensemble model (use five amino acid indices and different DWKNN engines with random K values). Secondly, the Sn and Sp values of both the base learner and the ensemble models are not very biased although the dataset is imbalanced. Thirdly, for the proposed methods the MCC values and Maximum MCC values are not very different, so there is no need to adjust the threshold values to go after the maximum, which has potential risks of over-fitting on the training dataset. Because when *N*_e_ = 4, the biggest MCC is obtained, so we choose it as the default hyperparameter value in the ensemble model.

**Table 1:** Performance comparisons between base learner and ensemble models on D92M over leave-one-out cross-validation. All the results are obtained by setting 0.5 as the default discrimination threshold to generate the prediction label except the Maximum MCC values which are obtained by identifying the thresholds that maximize the values of MCC

Metrics	Base learner	Ensemble model with different *N*_e_				
		2	4	6	8	10
Acc (%)	83.60	84.09	85.05	84.25	84.41	84.73
Sn (%)	81.42	80.31	81.1	80.47	80.63	80.94
Sp (%)	84.73	86.04	87.1	86.2	86.37	86.69
MCC	0.64	0.65	0.67	0.66	0.66	0.66
Maximum MCC	0.65	0.68	0.68	0.69	0.69	0.69

### Comparison with other methods

To demonstrate the performance of the proposed methods for predicting GPCR-drug interactions, we test them on the training dataset D92M and independent test dataset check390 respectively, and compare them with several state-of-the-art methods, including iGPCR-Drug, OET-KNN, QuickRBF, SVM, RF and RF + PPP. iGPCR-Drug employs PseAAC features of GPCRs, DFT features of drugs, and FKNN classifier. OET-KNN, QuickRBF, SVM, RF and RF + PPP employ PsePSSM features of GPCR sequences and DWT features of drug fingerprints. Beside classic machine learning modules, RF + PPP uses PPP to improve the prediction performance.

The results of leave-one-out cross-validation on D92M are listed in Table 2. It should be noted that the results of other six methods were reported in [35]. From this table we can see that the Sn values of the proposed methods are higher than the other methods, while the Sp, Acc and MCC values of the proposed methods are lower than the others. This may be due to the prediction engine used in the proposed methods is relatively weak.

**Table 2:** Performance comparisons of different methods on D92M over leave-one-out cross-validation. The best results for each metric are in bold

Method	Sn (%)	Sp (%)	Acc (%)	Str(%)	MCC
iGPCR-Drug	78.3	91.4	86.9	84.9	0.71
OET-KNN	77.8	88.7	85.0	83.3	0.67
QuickRBF	74.8	92.4	86.4	83.6	0.69
SVM	74.2	92.7	86.3	83.5	0.69
RF	76.5	**92.9**	87.3	84.7	0.71
RF + PPP	79.7	92.8	**88.3**	**86.3**	**0.73**
Proposed(Base learner)	**81.4**	84.7	83.6	83.1	0.64
Proposed(Ensemble model with *N*_e_ = 4)	81.1	87.1	85.1	84.1	0.67

For machine learning models, their generalization ability can be best evaluated through independent test. With D92M as training dataset, the results of independent test on check390 are listed in Table 3, in which the results of other six models are also from [35]. From this table we can find that the proposed methods almost always outperform the others across the five metrics, except OET-KNN, which achieves the highest value of Sp (84.2%) while having the lowest value of Sn (67.7%). In the other models, when only the classic machine learning methods are considered, RF characterized by advanced feature extraction and ensemble learning has the maximum MCC (0.54), which is ~ 13% lower than the proposed base learner (0.61), and ~ 16% lower than the proposed ensemble model (0.63). By employing complex PPP, RF + PPP get significant performance gains. However, the proposed methods (without PPP) outperform it across all metrics. All these results demonstrate the effectiveness of the proposed methods.

**Table 3:** Performance comparisons of different methods on the independent test dataset check390. The best results for each metric are in bold

Method	Sn (%)	Sp (%)	Acc (%)	Str(%)	MCC	Threshold
iGPCR-Drug	80.8	66.9	71.6	73.9	0.45	N/A
OET-KNN	67.7	**84.2**	78.7	76.9	0.52	0.5
QuickRBF	76.2	77.7	77.2	77.6	0.52	0.45
SVM	76.2	78.9	78.0	77.6	0.53	0.42
RF	78.5	78.1	78.2	78.3	0.54	0.51
RF + PPP	83.1	79.6	80.8	81.3	0.60	0.51
Proposed(Base learner)	**83.9**	80.0	81.3	81.9	0.61	0.5
Proposed(Ensemble model with *N*_e_ = 4)	83.1	82.7	**82.8**	**82.9**	**0.63**	0.5

## Discussion

GPCRs are the most important drug targets. The accurate identification of GPCR-drug interactions is fundamental to the discovery of GPCR-related drugs. Since the structure of GPCR is difficult to obtain, sequence-based machine learning methods are particularly important as an initial screening step to select the most likely candidates from hundreds or even thousands of candidates for wet-lab experiments, thereby reducing the cost and time of experiments. In this paper, a new sequence-based method is proposed for the determination of GPCR-drug interactions. Due to the usage of effective feature extraction method, the good prediction performance is achieved even with a relatively weak classifier as the prediction engine. Based on this basic method, the prediction performance can be further improved through ensemble learning.

In this paper, a new feature extraction method for GPCR sequences is proposed, which is inspired by the fact that the traditional BoW models are weak in extracting long fragment information (feature dimension is too high) and ignore the physicochemical properties of amino acids. It is shown through comparison experiments that the proposed feature extraction method precedes the traditional BoW models such as AAC and DPC. When compared with other models using PseAAC and PsePSSM, it is also competent. Although this method is used for GPCR feature extraction, it is clear that this method is a general peptide or protein feature extraction method that may be used to solve other target-drug interaction or protein classification problems. However, there are still three points need to be further investigated. First, there are hundreds of amino acid indices, and how to find the most proper ones is a big challenge. Second, the creation of workbooks relies on the clustering algorithm. In this study, only the simple C-means clustering algorithm is tried. More advanced clustering algorithms may create better wordbooks, so as to improve the performance of the model. Third, for fragments of different lengths how many clustering centers should be selected to constitute the dictionary entries? Intuitively, as the length of the fragments increases, there are more amino acid combinations, and the fragments should be grouped into more clusters. However, the theoretical optimum is difficult to obtain. In the future, we will do more in-depth research on this feature extraction method.

## Conclusions

In this paper, we propose a new sequence-based method for GPCR-drug interaction prediction. The remarkable feature of this method is to use a modified BoW model to represent GPCR sequences. Compared with the traditional BoW models, such as AAC, DPC, etc., this method can extract more pattern information from low order to high order, and restrict the dimension of feature space. In addition, the physicochemical properties of amino acids can be taken into account, so as to improve the representation ability. In terms of drug representation, we use the classical DFT transformation method. Compared with the original molecular fingerprint, DFT can extract more advanced pattern information, reduce the feature dimension and improve the prediction performance. The experimental results on the independent test dataset show that the proposed methods are better than the other sequence-based methods in generalization ability.

It should be noted that the proposed methods were tested on only one set of training and independent test dataset about GPCR-drug interaction, and they need to be evaluated on more datasets. We believe that although there are many effective feature extraction methods for amino acid sequences, such as AAC, DPC, PseAAC, PsePSSM, etc., the proposed new feature extraction method based on wordbook learning will also be useful to solve problems of target-drug interaction, protein-protein interaction, and protein attribute prediction.

## Methods

In this section, we explain the proposed method in detail, including the GPCR representation, drug representation and prediction engine. For sequence-based methods, the groundwork is to formulate the molecules with an effective mathematical expression that can truly reflect their innate relation with the label to be predicted [38]. When there is not enough data for automatic feature learning by neural network methods, BoW [39, 40] model is a quality replacement that is very flexible, and has been widely used in natural language processing and image processing. The first stage of BoW is to design the wordbook, for example, the n-gram method, which split the sentences (or sequences) into words with length n, and the set of unique words constitute the wordbook. This strategy has always been used in bioinformatics, such as AAC with *n* = 1, and DPC with *n* = 2. However, the power of AAC and DPC is very restricted, because the sequence order information is almost completely neglected. Increasing n can take more order information into account, but the size of the wordbook will be too large, for example, when *n* = 3, there are 20^3 unique words in the wordbook. This is a very high-dimensional and sparse representation that is harder to model for computational reasons (space and time complexity). Moreover, the physicochemical properties of 20 native amino acids are also ignored while these properties define the protein structures and functions [41]. To address these problems, we propose a novel method to represent GPCRs with BoW model, as described in the following passages.

### Design wordbook for GPCRs

A GPCR sequence containing *L* amino acid residues is often formulated in the following format, with the N-terminus at the left, and the C-terminus at the right.

1 $$ \mathrm{G}={\mathrm{R}}_1{\mathrm{R}}_2\dots \dots {\mathrm{R}}_{\mathrm{L}} $$

Given a physicochemical property of amino acids, the primary sequence can be encoded as a numerical sequence as follow,

2 $$ {\mathrm{G}}_{\mathrm{E}}={\mathrm{E}}_1{\mathrm{E}}_2\dots \dots {\mathrm{E}}_{\mathrm{L}} $$

where E_*i*_ is the property value of amino acid residue R_*i*_.

As described above, if we directly apply the n-gram model to split the GPCR sequences into words and select the unique ones to construct a wordbook, the wordbook size may be too large. To construct a small size wordbook, we will merge the similar words by clustering technology. Specifically speaking, if all the words are clustered into C clusters, then the C clustering centers constitute a small size wordbook because C is always much less than the number of unique words. The C-means clustering [42] method is used in this study.

The wordbook of GPCRs is created in the following steps:

1. Encode GPCR sequences according to a physicochemical property of amino acids.
2. Split the encoded sequences into fragments with different window sizes.
3. Cluster the fragments with the same length respectively, and take the clustering centers as the words of the GPCR wordbook.

**Fig. 5:**
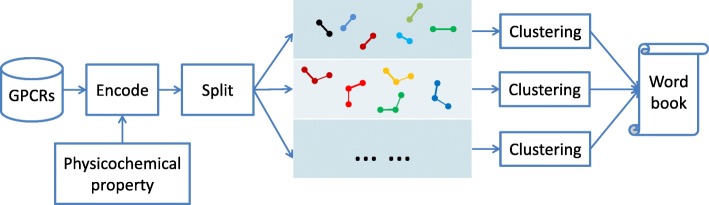
Flowchart of creating GPCR wordbook

The fragments can be sampled from the sequences in two modes. Sampling mode 1 is to sample fragments as many as possible when there are not enough sequences. In this case we can move the window along each sequence from the left to the right with stride 1. Sampling mode 2 is to randomly sample a certain amount of fragments from each sequence when there are sufficient sequences. Figure 5 illustrates the process of creating GPCR wordbook.

**Fig. 6:**
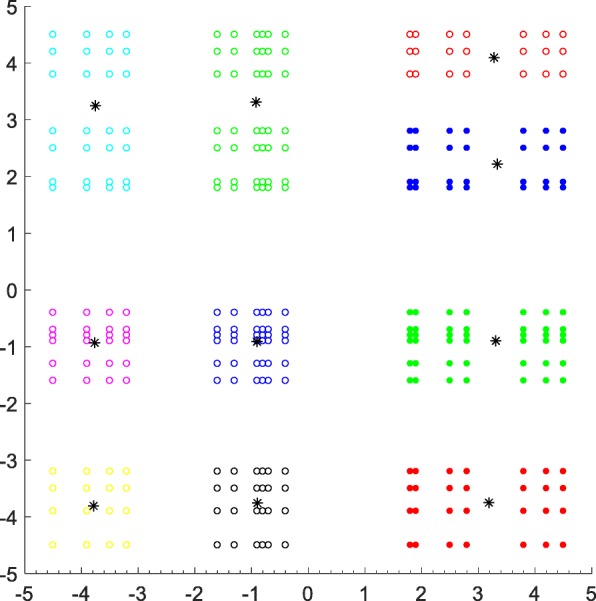
Fragments of length 2 sampled from the GPCR sequences encoded by hydropathy property. The sampled fragments belong to the same cluster are drawn in the same color and shape. The black asterisks are the clustering centers

In theory, any physicochemical property can be used here as long as it works in the interaction between GPCRs and drugs. The hydropathy is an important physicochemical property of amino acids and affects the structure, stability and basic properties of the proteins. We use the hydropathy property reported in [43] to encode the GPCRs, and then randomly select 500 fragments of length 2 from each encoded GPCR sequence. If the length of one sequence does not meet the condition, then sampling mode 1 is used. With the first element as the X-axis, and the second element as the Y-axis, then all the sampled fragments can be showed in Fig. 6, from which we can see that the fragments have obvious tendency of clustering. This indicates that it is reasonable to construct a smaller wordbook.

### Feature extraction from GPCRs

Based on the wordbook, any GPCR can be represented as a feature vector in the following steps:

(1) Encode the GPCR primary sequence by the same physicochemical property used in the process of creating wordbook.

(2) Split the encoded sequence into fragments of length *l* with sampling mode 1.

(3) Count the number of times each word appears in the sequence. If any fragment is closest to one word in the wordbook according to Euclidean distance, then we say that this word appear once.

(4) Formulate the GPCR as a feature vector containing the occurrence frequency of each word as follow:

3 $$ \mathbf{G}\left(l,{C}_l\right)=\left[{f}_1^l\kern0.5em {f}_2^l\kern0.5em \cdots \kern0.5em {f}_{C_l}^l\right] $$

where *l* is word length, *C*_*l*_ is the number of length-*l* words in the wordbook, and ${f}_i^l$ is the ratio between the number of the *i*th word and the number of fragments.

If we change the window size when splitting the sequence, then we can get more features so as to integrate more pattern information. Specially, it is difficult to cluster the fragments of length 1 due to no numerical stability, so we just use AAC for G(1,*C*_1_).

### Feature extraction from drugs

Molecular fingerprint is a way of encoding the structure of a molecule, and has been widely used in chemical informatics. Because of the effectiveness in previous work [21, 33, 35], we also extract features from drugs based on their molecular fingerprints. A drug’s MOL file is a chemical file format contains information about atoms and bond and can be obtained from KEGG database (https://www.genome.jp/kegg/) via drug code. Then this MOL file can be converted to molecular fingerprint through OpenBabel software (http://openbabel.org/). There are multiple output formats, such as FP2, FP3, FP4 and MACCS. In this study, the FP2 format that encodes a drug as a 256-bit hexadecimal string is used. If we regard the FP2 molecular fingerprint as a sequence of spaced samples by converting the hexadecimal char “0 ~ F” to number 0 ~ 15, then we can apply DFT on this digital signal.

DFT has been successfully used for the prediction of GPCR-drug interaction [33]. However, because the frequency amplitudes of a digital signal are in fact symmetrical, only the first 128 amplitudes are used here to make up the feature vector **D**.

4 $$ \mathbf{D}=\left[{A}_1\kern0.5em {A}_2\kern0.5em \cdots \kern0.5em {A}_{128}\right] $$

where *A*_*i*_ is the *i*th amplitude divided by the sum of all the 128 amplitudes.

### Representation of GPCR-drug pairs

Now we can represent the GPCR-drug pair denoted as **P** by concatenating the GPCR and drug feature vectors, i.e. **P** = **G**(*l*, *C*_*l*_) ⊕ **D**. For simplicity, we make *C*_*l*_ = 10*l. Because it is difficult to cluster the length-1 fragments due to no numerical stability, we just use the 20-dimensional AAC when *l* = 1. What’s more, to make the feature dimension of GPCRs equal that of drugs, we set *C*_4_ = 58. That is to say, *C*_*l*_ = 20, 20, 30, and 58 when *l* = 1, 2, 3 and 4 respectively. In such a way each pair is formulated as a 256D feature vector.

5 $$ \mathbf{P}=\left[{f}_1^1\kern0.5em \cdots \kern0.5em {f}_{20}^1\kern0.5em {f}_1^2\kern0.5em \cdots \kern0.5em {f}_{20}^2\kern0.5em {f}_1^3\kern0.5em \cdots \kern0.5em {f}_{30}^3\kern0.5em {f}_1^4\kern0.5em \cdots \kern0.5em {f}_{58}^4\kern0.5em {A}_1\kern0.5em {A}_2\kern0.5em \cdots \kern0.5em {A}_{128}\right] $$

### Prediction engine

We employ DWKNN classifier as the prediction engine, which have only one parameter and its performance depends on the feature representation to a great extent. DWKNN is an improvement on the original KNN algorithm. Its basic idea is to weight the evidence of the neighbor according to the distance from the unknown sample, and the smaller the distance is, the larger weight the neighbor will have.

When an unknown sample ***x*** is to be classified, the *K* nearest neighbors of ***x*** together with their class labels in the training dataset are given by $\left({\boldsymbol{x}}_k^{\ast },{\boldsymbol{y}}_k^{\ast}\right),1\le k\le K$. Let the distances of these neighbors from ***x*** be expressed as *d*_*k*_, which are ordered so that *d*_1_ ≤ *d*_2_ ≤ ⋯ ≤ *d*_*K*_, then the weight of the *k*th nearest neighbor can be defined as

6 $$ {w}_k=\left\{\begin{array}{l}\frac{d_K-{d}_k}{d_K-{d}_1},\kern0.6em {d}_K\ne {d}_1\\ {}1,\kern3.999998em {d}_K={d}_1\end{array}\right. $$

The Euclidean distance is used here, and it is clear that the smaller the distance of the neighbor is, the larger weight the neighbor will have. With the weights of neighbors, we can calculate the output of ***x*** as below,

7 $$ o=\raisebox{1ex}{$\sum \limits_{{\boldsymbol{y}}_k^{\ast }=1}{w}_k$}\!\left/ \!\raisebox{-1ex}{$\sum \limits_{k=1}^K{w}_k$}\right. $$

where ${y}_k^{\ast }=1$ indicates that the *k*th neighbor is a positive sample, i.e. interactive pair in this study. This output varies from 0 to 1, and can be taken as the probability of interaction. The larger the output is, the more likely that the query GPCR-drug pair is interactive. We usually choose a discrimination threshold *t* to generate the prediction label, for example, when *o* > *t*, we say the query sample is positive (interactive), otherwise, it is negative (non-interactive). This trick is very useful when the training dataset is imbalanced.

### Framework of the proposed methods

**Fig. 7:**
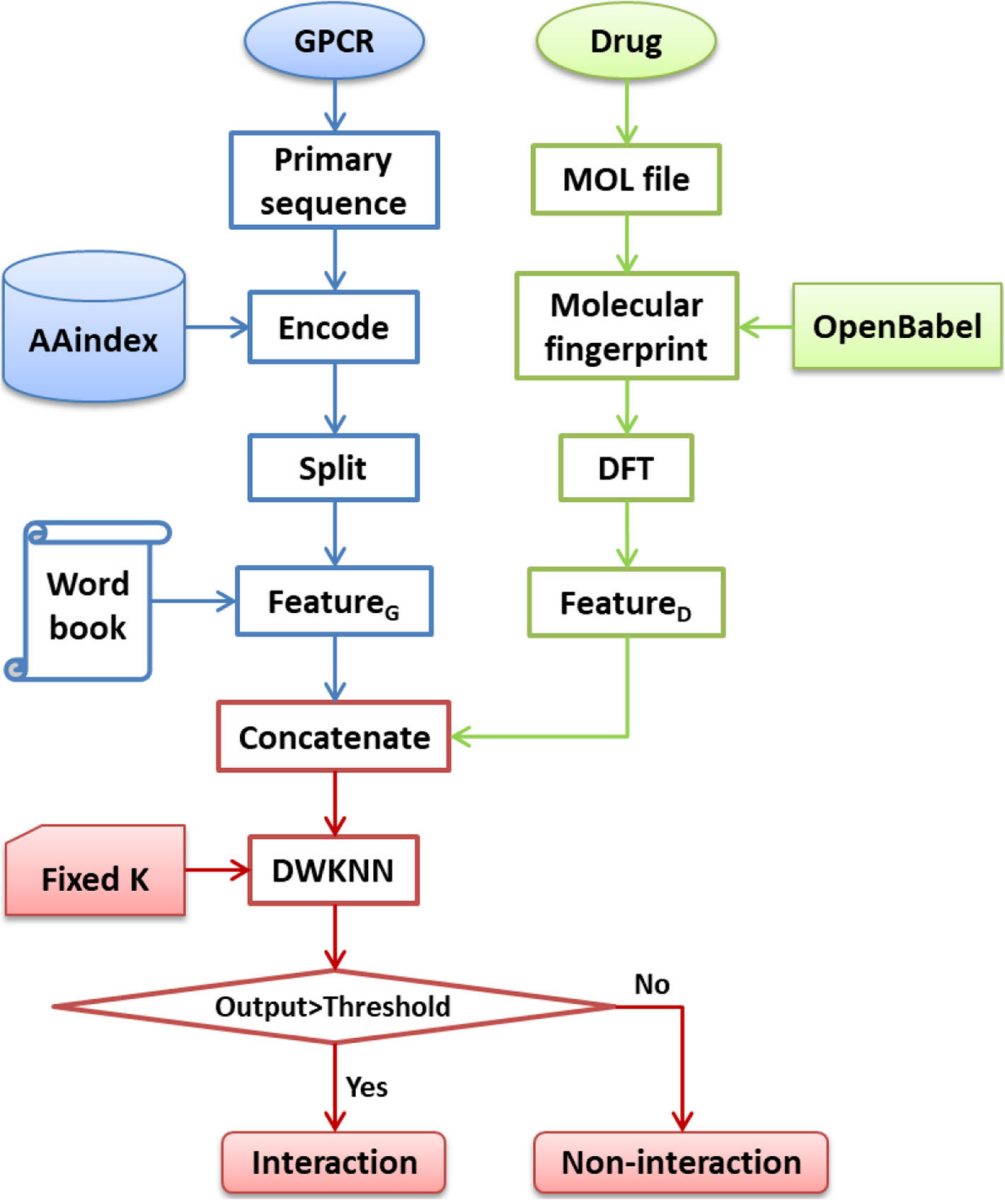
Framework of the proposed basic method

Figure 7 shows the framework of the proposed basic method. For a query GPCR-drug pair, we create the 128D feature vectors for GPCR (Feature_G_) and drug (Feature_D_) respectively, and then concatenate them into a 256D feature vector. This process is the same with that of creating training samples. The concatenated vector is input into the prediction engine DWKNN with a fixed K value (for example 13) to get an output, which is compared with a discrimination threshold (for example 0.5) to generate prediction label.

**Fig. 8:**
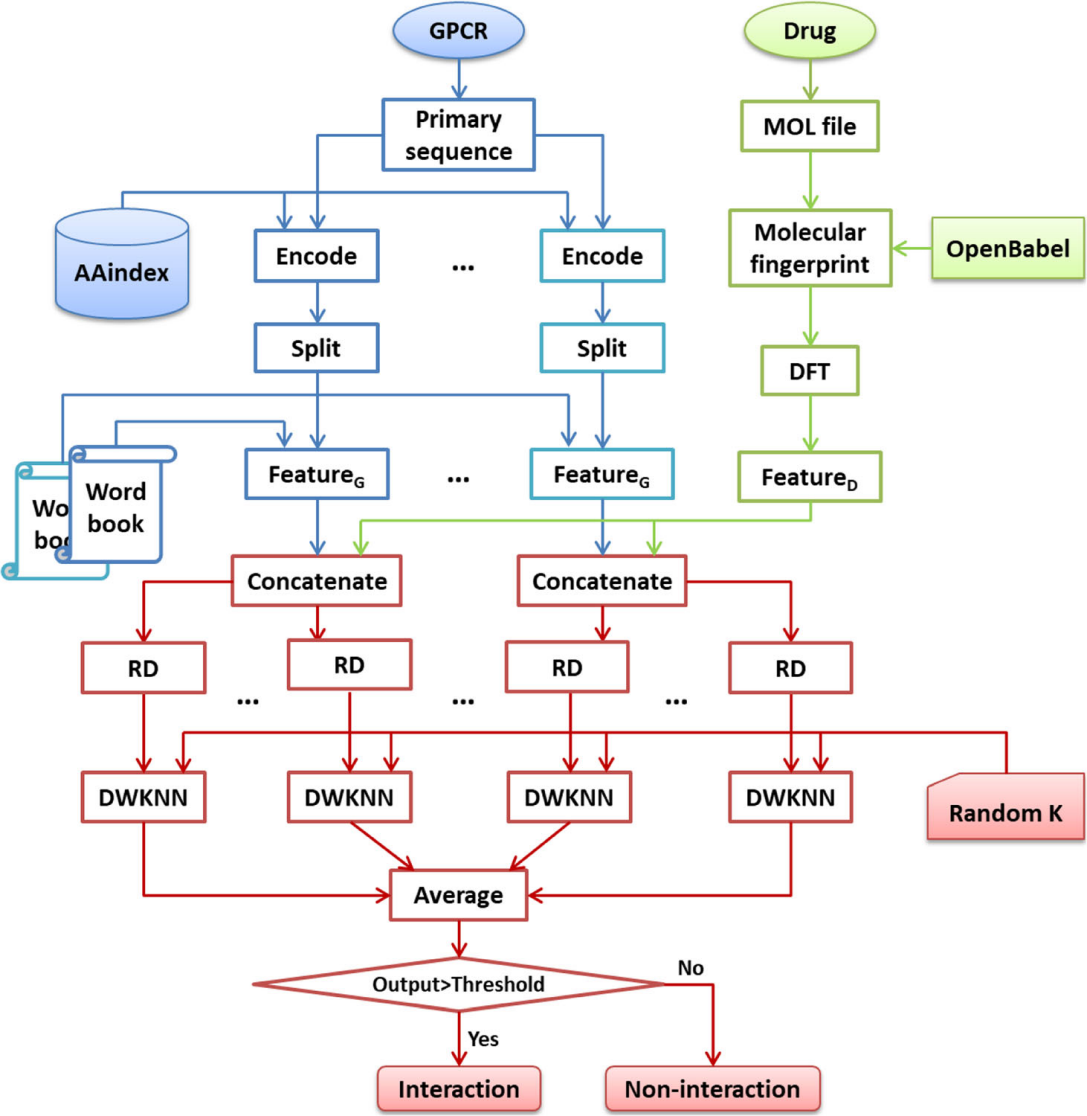
Framework of the proposed ensemble method

Figure 8 shows the framework of the proposed ensemble method, which can be described in the following steps: (1) different wordbooks are created with different amino acid indices respectively; (2) different kinds of Feature_G_ are extracted based on these wordbooks; (3) each kind of Feature_G_ is concatenated with Feature_D_; (4) to make the base learners as diverse as possible, the concatenated features are randomly discarded with a probability of 0.05 (RD), and then are input into different DWKNN engines with random K values sampled from 1 to 15; (5) the final output of the ensemble model is the average of the outputs of all base learners. It should be noted that the number of base learners depend on the number of amino acid indices and the number of prediction engines for each amino acid index (called *N*_e_). For example, if five amino acid indices are used and *N*_e_ = 2, then there will be 10 base learners in total. The proposed framework may be improved by some new techniques such as Optimal Bayesian Classification [44] and Bayesian Inverse Reinforcement Learning [45].

## Data Availability

The datasets used during the current study are available from http://202.119.84.36:3079/TargetGDrug/

## Abbreviations

AAC: Amino acid composition
AAindex: Amino acid index database
Acc: Accuracy
AUC: Area under an ROC curve
DPC: Dipeptide composition
BoW: Bag-of-words
DFT: Discrete Fourier transform
DWKNN: Distance-weighted K-nearest-neighbor
HTS: High-throughput screening
MCC: Matthews correlation coefficient
PseAAC: Pseudo amino acid composition
PsePSSM: Pseudo position specific scoring matrix
PPP: Post-processing procedure
RF: Random forest
ROC: Receiver operating characteristic
Sn: Sensitivity
Sp: Specificity
Str: The average of Sensitivity and Specificity
SVM: Support vector machine
